# Identifying Patient-Reported Care Experiences in Free-Text Survey Comments: Topic Modeling Study

**DOI:** 10.2196/63466

**Published:** 2025-02-24

**Authors:** Brian Steele, Paul Fairie, Kyle Kemp, Adam G D'Souza, Matthias Wilms, Maria Jose Santana

**Affiliations:** 1 Centre for Health Informatics, Cumming School of Medicine, University of Calgary, Cal Wenzel Precision Health Building, 3280 Hospital Dr NW, Calgary, AB, T2N 4Z6, Canada, 1 403-220-5110; 2 Department of Community Health Sciences, Cumming School of Medicine, University of Calgary, Calgary, AB, Canada; 3 Alberta SPORT SUPPORT Unit Patient Engagement Team, Calgary, AB, Canada; 4 Provincial Research Data Services, Alberta Health Services, Calgary, Alberta, Canada; 5 Department of Radiology, Cumming School of Medicine, University of Calgary, Calgary, AB, Canada; 6 Department of Pediatrics, Cumming School of Medicine, University of Calgary, Calgary, AB, Canada

**Keywords:** natural language processing, patient-reported experience, topic models, inpatient, artificial intelligence, AI, patient reported, feedback, survey, patient experiences, bidirectional encoder representations from transformers, BERT, sentiment analysis, pediatric caregivers, patient safety, safety

## Abstract

**Background:**

Patient-reported experience surveys allow administrators, clinicians, and researchers to quantify and improve health care by receiving feedback directly from patients. Existing research has focused primarily on quantitative analysis of survey items, but these measures may collect optional free-text comments. These comments can provide insights for health systems but may not be analyzed due to limited resources and the complexity of traditional textual analysis. However, advances in machine learning–based natural language processing provide opportunities to learn from this traditionally underused data source.

**Objective:**

This study aimed to apply natural language processing to model topics found in free-text comments of patient-reported experience surveys.

**Methods:**

Consumer Assessment of Healthcare Providers and Systems–derived patient experience surveys were collected and linked to administrative inpatient records by the provincial health services organization responsible for inpatient care. Unsupervised topic modeling with automated labeling was performed with BERTopic. Sentiment analysis was performed to further assist in topic description.

**Results:**

Between April 2016 and February 2020, 43.4% (43,522/100,272) adult patients and 46.9% (3501/7464) pediatric caregivers included free-text responses on completed patient experience surveys. Topic models identified 86 topics among adult survey responses and 35 topics among pediatric responses that included elements of care not currently surveyed by existing questionnaires. Frequent topics were generally positive.

**Conclusions:**

We found that with limited tuning, BERTopic identified care experience topics with interpretable automated labeling. Results are discussed in the context of person-centered care, patient safety, and health care quality improvement. Furthermore, we note the opportunity for the identification of temporal and site-specific trends as a method to identify patient care and safety concerns. As the use of patient experience measurement increases in health care, we discuss how machine learning can be leveraged to provide additional insight on patient experiences.

## Introduction

Patient-reported experience measurements (PREMs) offer opportunities to understand and improve the patient experience with the goal of ultimately improving health care. PREMs for inpatient experiences typically evaluate a single clinical encounter using survey items that assess different aspects of the care experience using Likert-type scales to determine the range of patient experiences on individual items (eg, “Did doctors treat you with respect?”) that are summed to domain-level composites (eg, overall experience with nurses, hospital environment, and discharge process) [1]. As the majority of questions on PREMs tend to use numerical scoring, the use of PREMs in health care quality improvement tends to focus on the tracking and improvement of quantitative scores. For example, regulations in the United States requires many hospitals to publicly report Hospital Consumer Assessment of Healthcare Providers and Systems (HCAHPS) scores [2]. However, a focus on quantitative summaries means that administrators, providers, and researchers could be missing out on experiences or concerns not being measured through existing quantitative domains [3-6].

In addition to the rating scales, patient experience surveys often include free-text comment responses where patients can share additional feedback and context on their experiences with health care services. These free-text comments can provide additional insight into patient experiences and how health care can be improved. Routine analysis of free-text comments may not be widely adopted for many reasons, possibly due to an unfamiliarity with methods for textual analysis and limited resources with which to conduct quality assurance in health care service settings [7,8].

In recent years, methodological developments in machine learning have enabled automated, efficient analysis of previously underused text data in health care. Natural language processing (NLP) refers to methods for representing text (and language) in ways that computers can understand and process. For example, researchers have used NLP methods with electronic medical records to predict clinical outcomes such as disease progression and health service utilization [9-11]. In the context of patient-reported outcome and experience measures, NLP could be applied to free-text comments to identify common themes and emerging concerns for specific hospitals or patient populations. Specifically, topic modeling can be used to identify distinct care experience topics and sentiment analysis can describe the emotional valence (positivity or negativity) of free text.

To date, few studies have applied NLP to patient experience data. In a systematic review, Khanbhai et al [12] identified 5 studies that used NLP to analyze routinely collected patient-reported measures, including the Press Ganey, HCAHPS, and 2 measures specific to experiences with cancer care. Furthermore, several studies applied NLP to nonstandard patient-reported experience data, such as forum or social media posts [12]. In our own literature review, we have identified 4 additional studies that have applied NLP to pre-existing, routinely collected patient experience measures [6,13-15]. We found that researchers have taken different approaches to topic modeling of patient-reported experience, including latent Dirichlet allocation (LDA), nonnegative matrix factorization, Top2Vec, and BERT (bidirectional encoder representations from transformers). Many of these studies trained new models or otherwise involved what could be considered a high degree of model tuning. Despite the promise of NLP methods for understanding patient experiences, the pace of innovation and the relative methodological complexity may present barriers to adoption in health care organizations. Organizations looking to implement routine analysis of open-ended text could benefit from knowing whether “off-the-shelf” pretrained topic models can provide useful insight without significant up-front costs. Furthermore, existing research on this topic focuses exclusively on the care experiences of adults. To address both gaps, the aim of this study was to (1) apply and evaluate unsupervised NLP with limited hyperparameter tuning to identify and describe topics found in the free-text comments of patient-reported experience surveys for hospital stays in Alberta, Canada, which cover (2) care experiences of adult and pediatric patients.

## Methods

This study relied on data collected by Alberta Health Services (AHS) as part of ongoing health care quality monitoring. At the time the study was conducted, AHS was the provider of inpatient health care in Alberta, a Canadian province with 4.7 million residents.

### Patient-Reported Experience Surveys

AHS uses 2 surveys to assess patient experiences with inpatient care—the Canadian Patient Experiences Survey on Inpatient Care (CPES-IC) survey and the Alberta Pediatric Inpatient Experience Survey (APIES). These surveys assess patient experience by asking questions on topics such as provider interactions (eg, doctors and nurses), the hospital environment (eg, noise levels and cleanliness), and the discharge process (eg, receiving written information). During the surveys, patients are also prompted for free-response comments (CPES-IC: “Is there anything else you would like to share with us about your hospital stay?”; APIES measure: “Is there something else you would like to share about your child’s and your family’s hospital experience? Please explain.”) which are recorded by the telephone operators.

The CPES-IC is a modified version of the HCAHPS, a survey commonly used in the United States to assess patient-reported experiences with inpatient care [1]. Details on the CPES-IC can be found at the Canadian Institute for Health Information website [16]. The APIES is a modified version of the Child HCAHPS, an extension of the HCAHPS designed to assess pediatric patient and family inpatient experiences [17]. While many questions are identical and the experience domains are comparable between the CPES-IC and the APIES, the pediatric survey has additional questions on experiences specific to the child and family experience. All questions used in this analysis are common between the CPES-IC, APIES, and the (Child) HCAHPS surveys. We used the date (year) of the survey, the respondent’s willingness to recommend a hospital (coded as most positive, “Definitely yes”), and the free-text comments. Responses were linked to administrative health data to provide descriptive statistics for the sample [18]. From this administrative data, we summarized admission category (urgent, elective), length of stay, sex, and age at discharge.

### Sampling Procedure

The survey sampling procedure is consistent between adult and pediatric populations. For each institution, AHS operators randomly dial patients (or caregivers of the same) meeting the eligibility criteria until 10% of the eligible discharges are included. Pediatric surveys were administered to eligible discharges from 16 institutions and adult surveys were administered to eligible discharges from 96 institutions. AHS makes multiple attempts to contact eligible patients. Data are collected exclusively via telephone. Respondents who agreed to answer the survey did so with the knowledge that these surveys are used for quality improvement and research purposes. More details on the sampling strategy and inclusion criteria have been published previously [19,20].

Textbox 1 describes the criteria for survey eligibility [20,21]. Patients were eligible to participate in the survey if they had a hospital stay lasting more than 24 hours, were not admitted to a psychiatric unit (or treated by a psychiatrist during inpatient stay), and did not die during the hospital stay. The eligibility criteria for pediatric inpatient visits are comparable. Caregivers (typically parents) of pediatric patients are contacted if pediatric patients meet the same conditions (for comparability with other jurisdictions, this study excludes pediatric surveys completed for newborn admissions with a length of stay less than 6 days).

Textbox 1. **Survey eligibility criteria.**

**Common eligibility criteria**

- Length of stay >24 hours.
- No psychiatric unit or psychiatrist on inpatient record for current stay.
- No death during inpatient stay.

**Specific eligibility: adult**

- Patient ≥18 years of age (at hospital discharge).
- Did not meet one of the conditions screened out on compassionate grounds (eg, still-births, babies tied to length of stay >6 days).

**Specific eligibility: pediatric**

- Patient <18 years of age (at hospital discharge).
- Caregiver not recorded as “no-publicity” (do not contact) in record.

### Analysis

**Figure 1.**
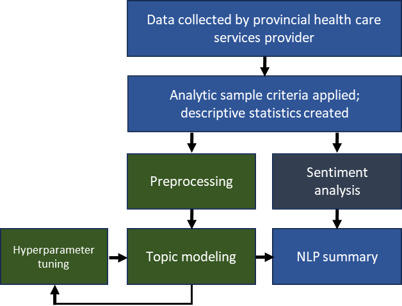
Analytic pipeline. NLP: natural language processing.

Figure 1 displays the analytic pipeline for this study. Data were linked by AHS Data and Analytics; data cleaning and descriptive statistics were conducted in SAS 9.4 and R (R Core Team; 4.2.1) [22]. After importing data into R and applying the analytic criteria (eg, date range and admission categories associated with newborns), white space was trimmed to identify and exclude any comments that may have contained only blank spaces. Additional checks based on comment length were used to check comment lengths and improperly coded missingness (eg, “N/A” instead of “”). Descriptive statistics were calculated for variables from patient experience surveys and linked administrative health data.

### Topic Modeling

Topic modeling was completed using the BERTopic package in Python [23]. BERTopic requires minimal preprocessing and accounts for semantic context by creating document embeddings and using a class-based term frequency-inverse document frequency method [23]. We used the default pretrained embeddings model (“all-MiniLM-L6-v2” from sentence-transformers). Other popular methods for topic modeling (such as LDA) focus on word frequency and do not account for the order and relationship of words [23,24]. Using embeddings-based models allow for comparisons with previous NLP PREMs work that have used Top2Vec and BERT-based models [6,14]. BERTopic can apply multiple representation models to generate multiple automated topic labels, allowing for a qualitative comparison of the interpretability of these topic labels.

The initial analytic process followed many of the suggested defaults from “best practices” in BERTopic documentation [25]. These included pre-extracting embeddings using a sentence transformer, removing English stopwords (common words), removing infrequent words (in this case, words used in fewer than 2 documents), setting the n-gram size to range between 1 and 2 words (identifying single words and 2-word terms), and setting a reproducible seed for the dimensionality reduction process. Before topic modeling, we removed duplicate comments. Each comment is assigned to a single topic. Topic labels (frequent words that describe a topic) are assigned by the model using representative terms. We did not provide any manual labeling at any stage in the topic modeling.

In the first model, we did not adjust parameters that would limit the number of potential topics beyond BERTopic’s automated topic reduction as a method for reducing outliers. In addition to the base representation, KeyBERTInspired was selected as an additional representation model backend with the hope of creating more interpretable topic labels.

To balance the competing priorities of creating topic representations with interpretable labels (eg, extracted keywords) and avoiding redundant topics, we tuned model hyperparameters to reduce the total number of topics. In the second iteration of our model, clustering parameters were adjusted to generate a smaller number of topics (minimum cluster size using Hierarchical Density-Based Spatial Clustering of Applications with Noise) and the term range was increased (n-gram: 2‐4). In the third (and final) model, term (n-gram) size was expanded to 1‐4. To compare additional automated topic labeling methods, PartOfSpeech and MarginalMaximumRelevance were added as representation models to complement existing base and KeyBERT topic representations.

Sentiment analysis was completed using the {sentimentr} package in R to calculate the free-text response length (in words) and sentence-averaged sentiments [26]. This package calculates sentence-level sentiments and evaluates linguistic context that modulates the positive or negative emotion (valence shifting). Specifically, sentimentr identifies polarized words in a sentence by referencing a sentiment dictionary, examines surrounding words for their relation (eg, negation and amplification), and weights the estimate of the sentiment accordingly. Sentiment strength is indicated by the absolute value of the sentiment score; positive and negative sentiment are indicated by positive or negative number. Neutral comments have sentiment scores closer to 0. Whole-comment sentiment was calculated to describe topics but were not incorporated into topic modeling.

To summarize the findings, we reported the 5 most frequently assigned, most positive, and most negative topics. We also summarize topics not currently measured on the patient experience surveys.

### Ethical Considerations

Research ethics approval was granted with a waiver of consent by the Health Research Ethics Board at the University of Calgary (adult experience survey approval REB22-0810; pediatric experience survey approval REB17-0769).

Records were linked and deidentified by AHS for privacy and confidentiality. Data were provided to the research team as part of a research agreement with AHS.

## Results

### Overview

Between April 2016 and March 2020, AHS collected 100,272 adult experience surveys and 7464 pediatric experience surveys that were linked to administrative data and provided to the researchers. Of these surveys, 43,522 (43.4%) adult surveys and 3501 (46.9%) pediatric surveys included free-text comments. Descriptive statistics are presented in Table S1 in Multimedia Appendix 1.

Most comments were short; 54% (23,503/43,522) of CPES-IC comments and 44.1% (1543/3501) of APIES comments were 25 words or less. After deduplication, the CPES-IC contained 94.6% (41,152/43,522) distinct comments and the APIES contained 99.1% (3471/3501) distinct comments.

Table 1 presents the number of topics per model. The final (third) model contained 86 CPES-IC topics and 35 APIES topics. As noted in the Methods section, hyperparameters were tuned to reduce the total number of topics and combine similar topics. Between models 1 and 2, there was a large reduction in the number of topics among the adult surveys (168 fewer topics; 27/195, 13.8% of model 1). The most frequent, positive, and negative topics were generally consistent between the 3 models; however, topics in the second model were relatively coarse in comparison with the other 2. Full results of the 3 models are presented in MultimediaAppendices 2  3.

**Table 1.** Topics per model. BERTopic-, KeyBERTInspired-, and MaximumMarginalRelevance-assigned labels were relatively consistent, PartOfSpeech labels were not assigned for any of the adult topics and many of the pediatric topics.

Model	CPES-IC^a^	APIES^b^
Model 1	195 topics (from 328)	29 topics (from 51)
Model 2	27 topics (from 86)	20 topics (from 35)
Model 3	86 topics (not reduced further)	35 topics (not reduced further)

a CPES-IC: Canadian Patient Experiences Survey on Inpatient Care.

b APIES: Alberta Pediatric Inpatient Experiences Survey.

### Model Topics

Tables S2-S4 in Multimedia Appendix 2 presents the most frequent topics (based on distinct comments).

Table S3 and Table S4 in Multimedia Appendix 2 present the most positive and most negative topics (based on the full sample). Below, we describe the topics according to model-extracted keywords.

### Topics – Adult Inpatient Stays

The most frequent topics for adult stays included positive experiences with multiple aspects of care (topic 1), positive and negative reports about food (topic 3), childbirth (topic 4), hospital cleanliness (topic 5), positive experiences with staff (topic 7) and overall stay (topics 2 and 6), the discharge process (topic 8), and noise (topic 9). Topic 10 appears to be a combination of several topics.

The most positive topics for adult stays included reported general satisfaction and positive experiences (topics 13, 81, 75, 23, 15, 79, 25, and 71), positive experiences with nursing staff (topic 76), and organized and efficient care (topic 86).

The most negative topics for adults included topics specific to hospital-acquired infection (topic 74), theft (topic 80), the admission process (topic 11), the hospital environment (temperature – topic 28; noise – topic 9), parking (topics 42 and 53), negative communication experiences with nursing staff (topic 45), and pain medication or medication mistakes (topic 20).

### Topics – Pediatric Inpatient Stays

The most frequent topics for pediatric stays included positive experiences with multiple elements of care (topic 1), the hospital (topic 3), nurses (topics 7 and 8), surgery (topic 9), and overall experiences (topic 10). Topic 6 was specific to pediatric hospitals. Topic 4 focused on rooms, including sleeping, shared rooms, and noise. Topic 5 focused on food and breastfeeding.

The most positive topics for pediatric surveys included excellent care and satisfaction (topics 35, 12, 23, 21, 10, 24, and 13), positive experiences with staff (topic 17), positive experiences with care for babies (topic 27). Topic 32 focused on care at the Stollery Children’s Hospital.

The most negative topics for pediatric surveys included those for the emergency room (topics 25 and 34), parking (topic 11), negative communication experiences with nursing staff (topic 16), needles and pain (topic 20), medication and pain (topic 2), hospital rooms (topic 4), the discharge process (topic 15), and food and breastfeeding (topic 5). Topic 31 appears to be a combination of several topics.

### Novel Topics

Here, we summarize identified topics that do not correspond to questions on the patient experience surveys (excluding topics for specific facilities).

Among the adult surveys, the final model identified birth and delivery (topic 4), ultrasounds and communication about diagnoses (topic 16), beds and mattresses (topic 18), surgery (topic 19), temperature (topic 28), facilities needing upgrades or funding (topic 30), desire for private rooms (topic 48), concerns about shared-between-gender rooms (topic 32), overcrowding (topic 85), perceptions of insufficient staffing levels (topics 34 and 73), needles and intravenous placement (topic 37), hip and knee replacements (topic 4), parking (topic 53), night or day shift staffing (topic 55), electronics or entertainment (topic 59; television, internet, or Wi-Fi), mobility or wheelchairs (topic 62), language and communication barriers (topic 64), physiotherapy (topic 67), hospital-acquired infection (topic 74), and theft (topic 80).

Among the pediatric surveys, the final model identified topics related to neonatal intensive care units or intensive care units (topic 19 and 33), social workers (topic 30), surgery (topic 9), parking (topic 11), and food and breastfeeding (topics 5 and 21).

## Discussion

### Principal Findings

In this paper, we applied easy-to-use and computationally efficient machine learning-based NLP to identify and describe patient-reported experiences in free-text survey responses. To the best of our knowledge, this work is the first to describe the application of topic modeling to routinely collected patient-reported experience measures among both adult and pediatric experience surveys. By following best-practices documentation, tuning parameters to prevent overlap and reduce the total number of topics, and applying automated labeling, this application of BERTopic identified experience topics not currently evaluated using existing survey instruments.

Many of the 10 most frequent topics were related to positive ratings of care (adult sample: 5/10, 50%; pediatric sample: 6/10, 60%). This is perhaps unsurprising, as most patients in Alberta report a high level of satisfaction [21,27]. In our analytic sample, we note that 72% (30,996/43,522) of adult survey respondents with comments would “definitely” recommend the facility to others and that the proportion was even higher (2837/3501 81%) among pediatric survey respondents with comments.

One of our main goals for this study was to create topic representations with interpretable automated labeling. Of the 4 representation models used to model topics, the topic labels were largely consistent between BERTopic, KeyBERTInspired, and MaximalMarginalRelevance. However, the PartOfSpeech method failed to apply labels to all the adult survey topics and many of the pediatric survey topics. As our comments are transcribed by operators, the comments may not represent natural speech and therefore may not have been suited to the default PartOfSpeech method [28]. Still, the consistency of labels among most of the topic labeling suggests that several methods can provide interpretable labeling with no manual classification.

Many distinct topics were generated among the CPES-IC respondents. The pediatric experience survey asks about needles and IVs, but we also found that they were also identified as a topic within adult experiences. Several topics concerned accommodations, with topics about shared between gender rooming, overcrowding, and desires for private rooms, as well as beds and mattresses, temperature, and electronics. Currently, the CPES-IC questions on the hospital environment only include those about noise and cleanliness. Patients also reported concerns about facilities and staffing, with topics noting that the facilities needed repair, perceptions of insufficient staffing levels, comparisons between day and night shift staffing, and concerns about language and communication barriers. We identified a single topic on hospital-acquired infection. Future studies could evaluate whether these topics could have relevance as part of broader indicators of patient safety.

APIES respondents are the parents or caregivers of pediatric patients and reflect the experiences and priorities of both the patient and their family. While many of the topics were common between adult and pediatric surveys (eg, reporting excellent care and communication experiences with health care providers), we identified topics related to intensive care, social workers, and food and breastfeeding. While 33% (1139/3501) of the pediatric patients surveyed were under the age of 1 (Table S1 in Multimedia Appendix 1), the specific needs of parents of newborns and nursing infants are not evaluated using the APIES survey. In addition, while the survey evaluates communication with health care providers, APIES does not evaluate whether the psychosocial needs of patients and families are being met while in hospital. The “social worker” topic identified in this analysis suggests opportunities for further evaluation.

In addition to the unique experience topics within each sample, we identified 2 categories of topics that were shared between adult and pediatric respondents—food and parking. The topics that focused on food may warrant further investigation and survey item development; whether patients are reporting experiences about the quality or options for those with dietary restrictions, meals are an important element of the patient’s experience. Previous research using other survey instruments have identified that parking is a relevant factor that affecting overall patient satisfaction [29,30]. Parking (or other topics related to transportation) may not be immediately actionable by administrators but could suggest issues with health care accessibility.

### Connection to Previous Work

Studies using more traditional qualitative methods have identified similar topics of care that are not assessed by Consumer Assessment of Healthcare Providers and Systems (CAHPS)–derived surveys. Nepal et al [5] conducted interviews with patients to identify priorities around inpatient experiences. Among the themes identified in the study, investigators noted that patients reported topics around privacy and room-sharing, cleanliness, food, and access to television. Furthermore, a study of patient comments on the Child HCAHPS (the analogue of APIES) identified several topics that closely mirrored those in our study, including “poor vein management/IV care,” “sleeping/comfort for parent/family,” “Hospital services/amenities (ie, food, parking),” “Security/safety issues,” and “physical/bodily privacy” [3]. An example comment in the manually classified “physical/body privacy” topic included the privacy to breastfeed [3].

There is also consistency between our work and other studies applying NLP to routinely collected PREMs. Gallan et al [6] applied topic modeling to a set of HCAHPS surveys with additional Press Ganey survey items that included multiple free-text response boxes. Whereas our summary of topics relied on the automated topic labels with representative words from each underlying representation model, Gallan et al [6] used top2vec followed by manual classification of multiple free-text comments. Despite our different topic modeling and labeling approach, the novel topics on the hospital environment identified in our study were aligned with those from their work (topics on “room temperature,” “bed and visitor comfort,” “facility and room design and condition,” and “roommate”). We identified a single study applying completely unsupervised models to routinely collected inpatient PREMs. Cammel et al [15] modeled topics with nonnegative matrix factor and used sentiment and topic frequency to describe and classify the priority of topics in 2 Dutch hospitals. Our results are not directly comparable as we focused on absolute values of positivity and negativity and the researchers explicitly reported the most frequent positive, negative, and neutral topics within each hospital. While the translation from Dutch to English may present challenges in comparison, we did note some overlapping topics with our own work (specifically, communication with staff, satisfaction with care, concerns about temperature, and perceptions of how busy the hospital was).

Recent work suggests that respondents who provide comments may be willing to share more feedback than what is currently collected. In a comparison with a sample of surveys with only single-item responses, Quigley and Predmore [4] reported that comments provided on the narrative items were more likely to contain actionable topics. These researchers also found that when multiple opportunities for free-text responses are offered as part of inpatient experience validation, a majority of parents provide more information. For researchers or providers interested in applying NLP in specific clinical settings, the development of multiple open-ended questions and validation with end users may be able to provide more actionable information [31]. For health systems currently administering CAHPS surveys, there are currently 2 sets of Narrative Item Sets (like those used by Quigley and Predmore [4]) that have been developed for use with ambulatory care and pediatric inpatient settings [32]. As Narrative Item Sets are developed for other are adopted among health systems administering CAHPS surveys, we anticipate that NLP methods will see further integration for monitoring patient experiences to drive quality improvement.

NLP can also be used to classify topics according to existing guidelines or themes. Ish et al [14] report on BERT and bag-of-words models to identify topics in CAHPS primary care surveys with free-text responses. Manual labeling of a training sample resulted in 26 potential classes. The researchers found that BERT outperformed the bag-of-words models, but note limitations based on the small sample size, complexity of topics, and potential actionability of these methods. Using a supervised approach based on a subset of manually labelled comments, Khanbhai et al [13] compared the performance of 6 machine learning models’ ability to classify free-text comment themes and sentiment from 2 questions assessing patient-reported experience in multiple care settings. None of their methods included embeddings-based methods (eg, BERT and Top2Vec), but the researchers found that a support vector machine approach had the highest accuracy. NLP may also enhance existing topics or themes in patient-reported experience data. Fairie et al [33] applied a novel label enhanced LDA method to categorize patient complaints and concerns submitted to a provincial health authority in Canada. This method used an existing manual (coarse) framework for comment classifications, LDA, and consensus by authors to determine topic labels. While the study is not directly comparable with our evaluations of topic novelty relative to existing survey questions, many topics in the concerns dataset were similar or identical to those identified in our work.

Patient-reported outcome measures (PROMs) can also contain free-text responses. We identified a single preprint that applied BERTopic to free-text comments from PROMs. Linton et al [34] used a weakly supervised text classification model on PROM comments to classify topics for health-related quality of life in colorectal cancer care. Using predefined themes and seed terms, the researchers compared 5 different topic models and found that BERTopic performed poorly on classification, relative to other methods. Experts (clinicians and scientists) who evaluated the results reported that the BERTopic-selected representative keywords were generally not relevant to the predefined themes, did not include the seed words, and that this differed from the keywords selected by other models. While the keywords applied to topics in our study were generally relevant, the work by Linton et al [34] suggests that researchers interested in applying NLP to patient-reported free text may need to evaluate multiple approaches for semisupervised topic modeling.

### Future Directions

This initial descriptive study explored how a fully unsupervised NLP model could identify novel topics. Based on the concordance of our results with previous literature, administrators and patient experience survey developers may benefit from developing survey items for future PREM. Several studies have applied semisupervised models by incorporating existing labels or themes based on operational needs or clinical relevance [13,14,33,34]. We could anticipate working more closely with patients, providers, and administrators to identify priorities and to guide NLP implementation.

As part of their evaluation of topic modeling for patient experience, Ish et al [14] discuss the issue of rare cases for training topic models. For example, the CPES-IC topic with the most obvious relevance for patient safety—the topic regarding hospital acquired infection—was classified in just 57 of the deduplicated comments. In this study, the focus on coarse topics reduces topic outliers but may prevent identification of rare but meaningful topics (eg, specific concerns on safety, communication, and hospital environment; elements of care most-relevant for specific clinical populations; or reasons for inpatient stays). In the model with the most finely detailed topics (for Model 1 for CPES-IC, refer to Multimedia Appendix 3), 71% (138/195) of topics were present in 50 or fewer deduplicated comments. Future studies could evaluate the relative frequency of topics by patient-population, hospital service, and institution as well as their relevance and actionability in these settings.

Beyond the identification of emerging patient-experience topics and integration into health care quality monitoring, comments may also assist in understanding patient care experiences. For example, a 2024 study with free-text Child HCAHPS comments manually coded for actionability and valence found that including these features as independent variables improved modeling overall experience outcomes [35]. Research also suggests that patient-reported experiences may be associated with unplanned readmission after discharge [19,36,37]. Future work could investigate how relevant survey items, comment topics, and sentiment may assist in understanding overall patient experiences and whether features assigned through NLP are associated with patient safety or readmission.

### Strengths and Limitations

This paper has several strengths. First, it is one of the first to apply NLP to a widely adopted patient-reported experience measure for both adult and pediatric patient settings. The use of NLP with routinely administered patient experience surveys presents the ability to incorporate patient comments into learning health systems [38]. Model hyperparameters (eg, n-gram size and cluster size) were adjusted only through recommendations in the best practices documentation, suggesting that a (relatively) off the shelf solutions can work without devoting significant resources to technological implementation. Second, the models were able to identify novel topics within single comments from existing surveys without additional data collection or instrument development (eg, narrative interviews and multiple open-ended questions) using an approach that requires computing resources already accessible inside most or all health systems. This suggests that health systems currently using CAHPS surveys can gain additional insight on patient experiences without implementing new survey methods. Finally, the application of topic modeling could assist in identifying health system–specific and patient population–specific elements of the patient experience. This study used surveys from a Canadian province with a publicly-funded health care system, but the findings are relevant for multiple health care system settings and countries. While CAHPS surveys were developed for use in the United States, CAHPS-derived surveys (those sharing the same or similar items) have been translated and adopted across several countries and clinical settings [39-43]. Our paper may contribute to larger efforts focused on identifying common elements of the patient-reported experience in multiple health care systems and settings.

There are also several limitations to the paper. This project aimed to identify and describe topics found in existing data and within a general population. We did not split the data to examine classification accuracy, nor did we examine applicability and relevance for patients or administrators. The majority of patients report positive in-hospital experiences on the survey and comments reflected those experiences, which likely introduced “noise” and prevented the identification of actionable topics. Uniquely actionable topics may not be identifiable within a general population approach; additional work is required to determine how these findings can translate to quality improvement efforts within specific patient subpopulations. While multiple topics may be discussed within a single patient comment, BERTopic will label these comments with a single classification based on predicted probabilities. The representation model-assigned keyword labels were generally interpretable, but other studies have found mixed results on their relevance [34]. Finally, measurement error was likely as comments were transcribed by telephone operators rather than by patients themselves [44].

### Conclusion

This paper provides the groundwork for future investigations within the Canadian context and beyond. It serves as both a descriptive report of observed topics and associated sentiments, but it also provides a technical proof-of-concept for researchers, policy makers, and administrators who may be looking for solutions to get the most out of patient-reported data using low-resource NLP approaches. We anticipate that partnering with patients and health care providers could help shape the development of future, semisupervised topic models with increased relevance for both patients and health systems.

## Supplementary material

10.2196/63466 Multimedia Appendix 1 Table S1: descriptive statistics.

10.2196/63466 Multimedia Appendix 2 Tables S2-S4: frequent, positive, and negative topics.

10.2196/63466 Multimedia Appendix 3 Full output for pediatric and adult surveys: models 1-3.

## Abbreviations

AHS: Alberta Health Services
APIES: Alberta Pediatric Inpatient Experiences Survey
BERT: bidirectional encoder representations from transformers
CAHPS: Consumer Assessment of Healthcare Providers and Systems
CPES-IC: Canadian Patient Experiences Survey on Inpatient Care
HCAHPS: Hospital Consumer Assessment of Healthcare Providers and Systems
LDA: latent Dirichlet allocation
NLP: natural language processing
PREM: patient-reported experience measure
PROM: patient-reported outcome measure
